# A signal detection method for temporal variation of adverse effect with vaccine adverse event reporting system data

**DOI:** 10.1186/s12911-017-0472-y

**Published:** 2017-07-05

**Authors:** Yi Cai, Jingcheng Du, Jing Huang, Susan S. Ellenberg, Sean Hennessy, Cui Tao, Yong Chen

**Affiliations:** 1 Pieces Technology, 8435 N Stemmons Fwy #1150, Dallas, TX USA; 2 School of Biomedical Informatics, University of Texas Health Science Center at Houston, Houston, TX USA; 3 Perelman School of Medicine, University of Pennsylvania, Philadelphia, PA USA

**Keywords:** Heterogeneity testing, Signal detection, Vaccine Adverse Event Reporting System (VAERS)

## Abstract

**Background:**

To identify safety signals by manual review of individual report in large surveillance databases is time consuming; such an approach is very unlikely to reveal complex relationships between medications and adverse events. Since the late 1990s, efforts have been made to develop data mining tools to systematically and automatically search for safety signals in surveillance databases. Influenza vaccines present special challenges to safety surveillance because the vaccine changes every year in response to the influenza strains predicted to be prevalent that year. Therefore, it may be expected that reporting rates of adverse events following flu vaccines (number of reports for a specific vaccine-event combination/number of reports for all vaccine-event combinations) may vary substantially across reporting years. Current surveillance methods seldom consider these variations in signal detection, and reports from different years are typically collapsed together to conduct safety analyses. However, merging reports from different years ignores the potential heterogeneity of reporting rates across years and may miss important safety signals.

**Method:**

Reports of adverse events between years 1990 to 2013 were extracted from the Vaccine Adverse Event Reporting System (VAERS) database and formatted into a three-dimensional data array with types of vaccine, groups of adverse events and reporting time as the three dimensions. We propose a random effects model to test the heterogeneity of reporting rates for a given vaccine-event combination across reporting years. The proposed method provides a rigorous statistical procedure to detect differences of reporting rates among years. We also introduce a new visualization tool to summarize the result of the proposed method when applied to multiple vaccine-adverse event combinations.

**Result:**

We applied the proposed method to detect safety signals of FLU3, an influenza vaccine containing three flu strains, in the VAERS database. We showed that it had high statistical power to detect the variation in reporting rates across years. The identified vaccine-event combinations with significant different reporting rates over years suggested potential safety issues due to changes in vaccines which require further investigation.

**Conclusion:**

We developed a statistical model to detect safety signals arising from heterogeneity of reporting rates of a given vaccine-event combinations across reporting years. This method detects variation in reporting rates over years with high power. The temporal trend of reporting rate across years may reveal the impact of vaccine update on occurrence of adverse events and provide evidence for further investigations.

## Background

The adverse effect (AE) of a medication is a broad term referring to unwanted, uncomfortable, or dangerous effects that a medication may have [1]. To monitor the safety of drugs/vaccines, the Centers for Disease Control and Prevention (CDC) and the Food and Drug Administration (FDA) maintain post-marketing surveillance programs of adverse event reports in association with drugs/vaccines^2^. The Vaccine Adverse Event Reporting System (VAERS) is a national vaccine safety surveillance program which contains reports received from 1990 to present [2]. VAERS accepts spontaneous reports from vaccine manufacturers, health care professionals and vaccine recipients [3]. The VAERS report collects information on the administered vaccines, the experienced AE, age, gender, and recovery status. Because any reported event following vaccination represents a temporal but not necessarily causal association, these reports are generally thought to provide weak evidence for a causal effect (although there are exceptions). Therefore, databases of the safety surveillance program are used to identify signals of potential AEs, with further investigation necessary to determine causality. Public health officials increasingly use data mining approaches to aid in the identification of signals that might otherwise be missed because of the large volume of reports (approximately 30,000 reports per year) [3].

Disproportionality measures are commonly used to identify safety signals in surveillance database. These methods compare the observed count for a vaccine-event combination with an “expected” count under the assumption that there is no causal association between the vaccine and AE. To apply the methods, a vaccine-AE matrix with types of vaccine as the column variable and types of AE as the row variable, respectively, is constructed. Each cell of the matrix is the count of reported events for the corresponding vaccine-AE combination. These approaches focus on identifying the AEs with higher reporting rates (number of report for a specific vaccine/number of reports for all vaccines) for a specific vaccine or identifying the vaccines associated with higher reporting rates of a specific AE. These methods include frequentist approaches such as Proportional reporting ratios^3^, Reporting Odds Ratios [4], Chi-squared test of independence [5] and Maximum Likelihood Ratio test [6] and the Bayesian approaches such as Multi-item Gamma Poisson Shrinker [7] and Bayesian Confidence propagation Neutral Network [8]. All of the aforementioned methods identify safety signals by calculating a score and comparing it to a pre-specified threshold.

The VAERS database has an important feature: the reports have been collected since 1990. Most of the existing methods collapse all the reports from different years together without considering the possibility of a temporal trend in the reporting rate. However, ignoring the heterogeneity or temporal trend of reporting rates across years may miss important signals. This is a particular concern for flu vaccines, which change every year to match the strain of flu virus expected to be prevalent [9–11]. The alteration of a vaccine may change its safety profile over time. Data from VAERS can be used to detect signals of changing safety profile for a vaccine over time. In this study, we extracted the counts of reports for each vaccine type *in each year* and reconstructed the reporting rates data in VAERS with respect to a vaccine-AE combination by constructing a *three-dimensional data array* with types of vaccine, types of adverse events and reporting time as the three dimensions. With this newly constructed dataset, we have a unique opportunity to identify significant variation in reporting rates across years by testing the null hypothesis that the reporting rates for each year are the same. We propose a rigorous statistical model and a powerful testing procedure for signal detection of temporal variation in AE reporting using VAERS data.

## Method

### Data resource and exaction

We applied our method to the FLU-3 vaccine, which is a synthetic trivalent vaccine consisting of three inactivated influenza viruses: two different influenza type A strains and one influenza type B strain. The influenza vaccine is produced by multiple manufacturers each year and updated annually to include the viruses that will most likely circulate in the upcoming season [12, 13]. We first downloaded all VAERS data from the VAERS website and imported it to our local MySQL server. We then searched for and extracted all serious FLU3 vaccine-AE reports (i.e., death, life-threatening illness, hospitalization, prolonged hospitalization, or permanent disability) from 1990 to 2013. Trivalent influenza vaccine is formulated annually, based on influenza strains projected to be prevalent in the upcoming flu season [14].

VAERS uses MedDRA (Medical Dictionary for Regulatory Activities) to categorize reported AEs. The MedDRA terminology is the international medical terminology developed under the auspices of the International Conference on Harmonisation of Technical Requirements for Registration of Pharmaceuticals for Human use [15]. It has a five-level structural hierarchy: from Lowest Level Term, Preferred Term (PT), High Level Term, High Level Group Term, to System Organ Class (SOC). A PT is a distinct descriptor for single medical concept like a symptom, sign, disease, and etc. A SOC is the highest level of the hierarchy that provides the broadest concept for data retrieval. MedDRA now has more than 21,000 PT terms and 26 different SOCs and each PT is linked to at least one SOC [13]. In the VAERS database, each report is manually assigned a MedDRA term by clinical experts [16].

In order to facilitate further statistical analyses, we grouped the large number of PTs in MedDRA to the SOC levels. Each PT term can be associated with multiple SOCs. To avoid double counting, we needed to determine the primary SOC for each PT. The rules to assign a primary SOC to a PT terms according to the MedDRA guideline [17] can be complicated, usually involving expert manual reviews that can be very time consuming. To simply this process, we first retrieved all the SOCs that a PT linked to by using National Center for Medical Ontology (NCBO) web services [18]. We then assigned a primary SOC to the PT term based on the SOC International Agreed Order, as shown in Table 1. By doing that, each PT term had one primary SOC. We then considered the AEs on the SOC level for further analysis.

**Table 1:** International Agreed Orders of SOCs

SOC	Order	SOC	Order
Infections and infestations	1	Gastrointestinal disorders	14
Neoplasms benign, malignant and unspecified (inccysts and polyps)	2	Hepatobiliary disorders	15
Blood and lymphatic system disorders	3	Skin and subcutaneous tissue disorders	16
Immune system disorders	4	Musculoskeletal and connective tissue disorders	17
Endocrine disorders	5	Renal and urinary disorders	18
Metabolism and nutrition disorders	6	Pregnancy, puerperium and perinatal conditions	19
Psychiatric disorders	7	Reproductive system and breast disorders	20
Nervous system disorders	8	Congenital, familial and genetic disorders	21
Eye disorders	9	General disorders and administration site conditions	22
Ear and labyrinth disorders	10	Investigations	23
Cardiac disorders	11	Injury, poisoning and procedural complications	24
Vascular disorders	12	Surgical and medical procedures	25
Respiratory, thoracic and mediastinal disorders	13	Social circumstances	26

### Statistical Method

The surveillance data of FLU3 vaccine were structured in a table format with vaccine-AE combination being the column variable, and reporting year being the row variable, as shown in Table 2. Therefore, the dataset contained 24 rows and 26 columns in total. The entries in the table cell were ${n}_{ij}$, defined as the number of events reported for the ${j}_{th}$ vaccine-AE combination during the ${i}_{th}$ year. The total number of reported cases for all the vaccine –AE combinations in ${i}_{th}$ year was denoted as ${n}_{i.}$, the total number of ${j}_{th}$ vaccine-AE combinations for all the years was denoted as ${n}_{. j}$, and the total number of events, which was the grand total of the table, was denoted as ${n}_{..}$. For a given ($i$, $j$), the number of reports in other years and in other vaccine-AE combinations can be summarized concisely by a simple 2 × 2 table as follows.

**Table 2:** Data structure of numbers of reports with respect to a given vaccine-AE combination

	j-th Vaccine- AE	Other combinations	
i-th Year	${n}_{ij}$	${n}_{i.}$. - ${n}_{ij}$	${n}_{i.}$.
Other years	${n}_{. j}-{n}_{ij}$	$\Big({n}_{..}-{n}_{i.\Big)}-\left({n}_{. j}-{n}_{i j}\right)$	${n}_{..}-{n}_{i.}$
	${n}_{. j}$	${n}_{..}-{n}_{. j}$	${n}_{..}$

For a given vaccine-AE combination (*j*), the number of reports in the ${i}_{th}$ year was assumed to follow a Poisson distribution: ${n}_{i j}\sim Poisson\left({n}_{i.}\times {p}_{i j}\right)$ where ${p}_{ij}$ was the reporting rate and the parameter of interest. To test for the heterogeneity of reporting rates of a given vaccine-AE combination across years, we focused on the data for a fixed vaccine-AE combination for each analysis, thus we suppress the notation $j$ from now on. In order to test for the heterogeneity of reporting rates from 1990 to 2013, we assumed a random effects model for the reporting rates across years, such that $logit\left({p}_i\right)\sim N\left({\beta}_0,{\tau}^2\right)$ and ${\beta}_0= logit\left({p}_0\right)$, where the parameter ${p}_0$ is the overall reporting proportion across all the year and the parameter ${\tau}^2$ represents the variation in reporting rates across the 24 reporting years. When ${\tau}^2$ is close to zero, the reporting rates are roughly the same for each year and equal to the overall reporting rate ${p}_0$. On the other hand, when ${\tau}^2$ is “large”, there is a substantial variation in reporting rates across years, which may be an important signal in vaccine safety. In the following section, we propose a rigorous statistical test to identify and test for such a “large” variation.

The proposed testing procedure is formulated as a three-stage procedure to test the variance of year-specific reporting rate, i.e., H_o_: ${\tau}^2=0$. Specifically,

i. Calculate the likelihood of the vaccine-AE combination under ${\tau}^2=0$:
  $$ {L}_0\left({p}_0\right)= Pr\left({n}_{i j}\Big|{n}_{i.};{p}_0\right) = {\displaystyle \prod_{i=1}^I}\left( exp\left(-{n}_{i.}{p}_0\right)\times {\left({n}_{i.}{p}_0\right)}^{n_i}\right)/\left({n}_i!\right). $$
  The estimation of the parameter ${\widehat{p}}_0$ is obtained by maximizing the likelihood ${L}_0\left({p}_0\right)$.
ii. Calculate the likelihood of the vaccine-AE combination under ${\tau}^2\ne 0$:
  $$ {L}_a\left({\beta}_0,{\tau}^2\right)= Pr\left({n}_{i j}\Big|{n}_{i.};{\beta}_0,{\tau}^2\right) = {\displaystyle \prod_{i=1}^I}{\displaystyle \underset{0}{\overset{1}{\int }}}\frac{exp\left(-{n}_{i.}{p}_i\right)\times {\left({n}_{i.}{p}_i\right)}^{n_i}}{n_i!}\times \frac{ \exp \left(-\frac{{\left( logit\left({p}_i\right)-{\beta}_0\right)}^2}{2{\tau}^2}\right)}{p_{i j}\left(1-{p}_i\right)\tau \sqrt{2\pi}} d{p}_i. $$
  The estimation of the parameters ${\widehat{\beta}}_0,{\widehat{\tau}}^2$ is obtained by maximizing the likelihood ${L}_a\left({\beta}_0,{\tau}^2\right)$.
iii. Obtain the likelihood ratio test (LRT) statistic by plugging in the estimation of the parameters to the likelihoods ${L}_0$ and ${L}_a$ in Eq. (1). The *p*-value of this test is obtained by compare $L R T$ to the chi-square distribution with one degree of freedom.
  1 $$ L R T = \frac{L_a\left({\widehat{\beta}}_0,{\widehat{\tau}}^2\right)}{L_0\left({\widehat{p}}_0\right)} $$

The data suggests evidence of temporal variations in reporting rates for the vaccine-AE combination across years if the test statistic $L R T$ is greater than the threshold of a significant *p*-value (e.g., *p* < 0.05). When several vaccine-AE combinations are considered, a Bonferroni-type correction can be used to control for the overall Type I errors.

**Fig. 1:**
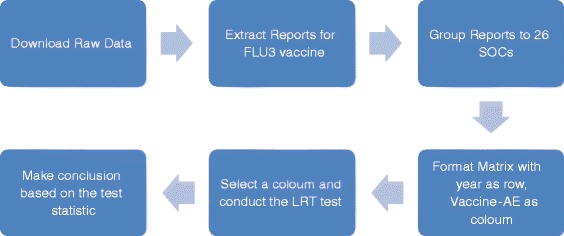
Flowchart of the proposed test process

The main procedure of the test is summarized in Fig. 1.

An important advantage of the proposed random effects model is that testing for the variation is equivalent to testing a single parameter ${\tau}^2=0$, which has only 1° of freedom. Such a procedure is much more powerful than a fixed effects model, which requires testing multiple parameters ${p}_1={p}_2=\dots ={p}_{24}$, with 24 - 1 = 23° of freedom. This LRT test is expected to be powerful because it is the well-known heterogeneity test in the variance component model [19, 20]. As a technical note, under the null hypothesis, the parameter ${\tau}^2$ lies on the boundary of its parameter space [0, ∞). Special considerations may be needed to account for such a boundary constraint [21, 22]. Here we choose to use the chi-square distribution with one degree of freedom, in order to keep the procedure simple and conservative. As we will illustrate later, this test can effectively identify signals in variation of reporting rates.

## Results

We applied the proposed LRT test to the 26 SOC types of AEs reported for vaccine FLU3 to detect significant variation in reporting rates over years for each of the 26 vaccine-AE combinations. For example, we tested the null hypothesis that the reporting rate of SOC1 (infections and infestation) after vaccination is the same from year 1990 to 2013. For each of the 26 FLU3-SOC combinations; we applied the proposed LRT and obtained the *p*-values. The 26 *p*-values are categorized into three categories based on the magnitude: less than 0.001, between 0.001 and 0.05, larger than 0.05. The counts of AEs, the LRT test statistics and the *p*-values of the LRT test for three selected FLU3-SOC combinations in each category are summarized in Table 3.

**Table 3:**
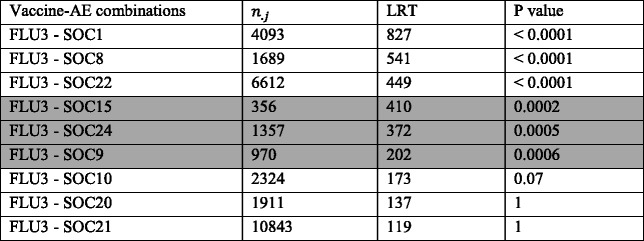
Number of reports, LRT test statistics and *p*-value for three selected FLU3-SOC combinations in each of the three *p*-value categories (*p* < 0.0001, 0.001 < *p* < 0.05, and p > 0.05)

**Fig. 2:**
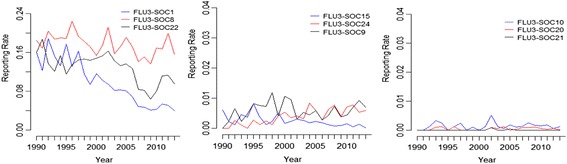
Trajectories of estimated reporting rates over time for selected FLU3–SOC combinations categorized by magnitude of *p*-values of the LRT test. The left panel contains the trajectories of reporting rates for FLU3-SOC combinations with *p*-value less than 0.0001. The middle panel contains the combinations with *p*-value between 0.0001 and 0.05. The right panel contains the combinations with *p*-value larger than 0.05

Figure 2 presents the trajectories of the reporting rates from year 1990 to 2013 for the FLU3-SOC combinations categorized by the magnitude of *p*-values of the LRT test. The left panel shows the reporting rates of three FLU3-SOC combinations with *p*-values less than 0.001. The reporting rates substantially fluctuate across reporting years compared to the reporting rates in the other two panels. As shown in the plot, SOC1 (infections and infestations) has a clear decreasing trend across years. The middle panel shows three FLU3-SOC combinations with the *p*-value between 0.001 and 0.05. The variation in the reporting rates are less obvious compared to the reporting rates in the left panel but more obvious than those in the right panel. The right panel shows the trajectories of reporting rates for three FLU3-SOC combinations which are homogeneous across years according to the LRT test. The change of the reporting rates across years is very small. There are zero observations for SOC20 and SOC21 in some of the reporting years. The visualized trends of reporting rates over years are consistent with our findings using the LRT test.

**Fig. 3:**
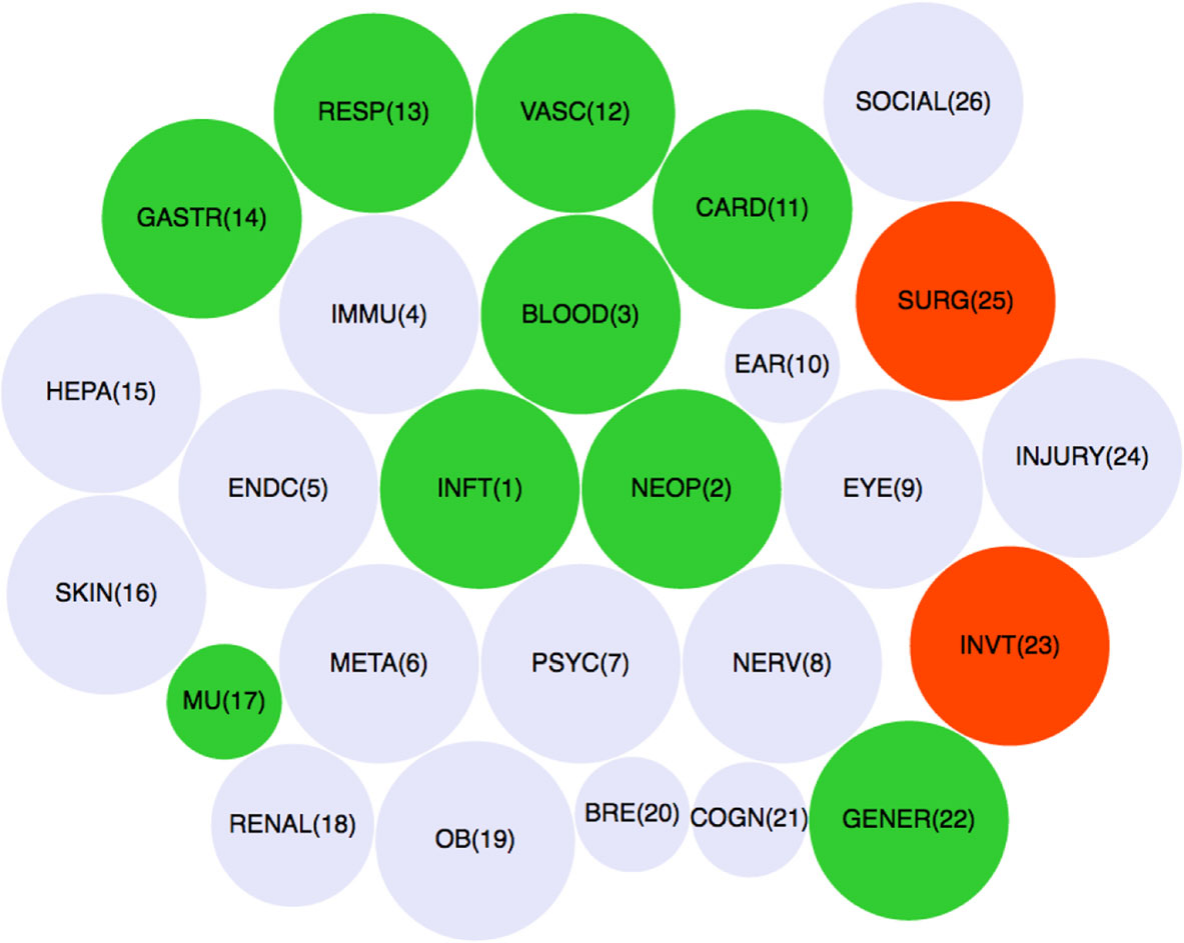
Bubble plot of LRT test result for FLU3-SOC combinations. The largest bubble stands for the SOC with *p*-value less than 0.001, the median size bubble stands for the SOC with *p*-value between 0.001 and 0.05, the smallest bubble size denoting the SOC with *p*-value large than 0.05. The bubble in *red* indicates the combinations with increasing reporting rates over time. The bubble in *green* indicates the combination with decreasing reporting rates trend. The bubble in light *blue* indicates the combination with unobvious reporting rates trend

In order to better present the results, we also developed an innovative visualization tool to concisely summarize the results from the LRT tests for all the 26 FLU3-SOC combinations. Specifically, the bubble plot in Figure 3 shows the size of the *p*-values from the LRT test and *the temporal trend* of each FLU3-SOC combination. The order of the SOCs (SOC1-SOC26) was based upon the determination of the International Conference on Harmonization Expert Working Group for MedDRA, according to MedDRA Guide Version 19.1 [16]. The size of the bubble reflects the level of heterogeneity of the reporting rates across years, as indicated by the categories of *p*-values. Figure 3 suggests that some SOCs, such as infection and infestations, blood system disorders, immune system disorders, endocrine disorders, Metabolism and nutrition disorders, have a high level of variation in reporting rates across years. The temporal trend of the FLU3-SOC combinations is indicated by color, with red denoting an increasing trend of the reporting rate, green denoting a decreasing trend of reporting rate, and light blue denoting an ambiguous trend of reporting rate.

## Discussion

In this study, we developed a random effects model with a likelihood ratio test to detect the heterogeneity of reporting rates of vaccine and AE combinations over time. We applied our method to the FLU3 vaccine to detect the AEs with heterogeneous reporting rates across years. The findings are consistent with the estimated reporting rates for each year. To the best of our knowledge, few methods have been developed to reveal the temporal trend of AE reporting rate. The sharp increasing or decreasing of reporting rate at a certain year may be associated with changes of virus strain and vaccine ingredients. Our method can be used to detect significant differences in reporting rates over time and provide signals requiring further investigation.

Our method can be used to make further investigations on a specific type of PT when heterogeneity of reporting rates of specific vaccine-SOC combinations is detected, since each SOC level is linked to many PTs. Furthermore, as a data mining tool to systematically detect safety signals, our method can be applied to other surveillance databases such as the Adverse Event Reporting System (AERS) administered by FDA for drug adverse events. When heterogeneity of reporting rates of a given drug/vaccine-AE combination is found, it suggests the need to monitor the temporal trend of the drug/vaccine-AE reporting rate and it warrants development of new statistical models to model and predict the temporal trend in the future.

We faced two major statistical challenges in this study. The first is the large number of zero cells in the data matrix. This is a common feature for the large safety databases used for post-market surveillance. In the VAERS database, with more than 4000 types of AEs, the percentage of vaccine-AE combinations with more than 90% of observed zero-count cells is as large as 88%. The percentage of zero values ranges from 57 to 99%. To take into account such high percentages of observed zero cells, we can extend our method by using a zero-inflated Poisson distribution to model the reporting rate. The second is that the likelihood function constructed in our test is not a genuine likelihood function, because the marginal densities of ${n}_{ij}$ are multiplied together without accounting for the correlation among them. Such a pseudolikelihood function is called a composite likelihood function, which can be constructed by multiplying (weighted) marginal or conditional densities together even when they may not be independent [23–25]. Therefore, the likelihood ratio test, $L R T$, is a composite likelihood ratio test. Chen and Liang [22] have derived the asymptotic distribution of the composite likelihood ratio test when one of the parameters is on boundary, which is a mixture of a Chi-squared distribution with zero degrees of freedom and a weighted Chi-squared distribution with one degree of freedom. The calculation of such a mixture distribution is very complicated and need to be carried out for each AE-combination. In this paper, we adopt the simple Chi-squared test with one degree of freedom.

Notably, our approach is intended to filter out weak safety signals and identify the more important ones that might merit further investigations. However, this method cannot be used as a significance test to quantify the strength of the evidence of heterogeneity. The magnitude of the *p*-value obtained from the test should not be over interpreted. It suggests statistical significance which is not an indicator of the importance of the evidence. Larger sample size can achieve higher statistical significance, but the clinically importance of the results should be discussed with domain experts.

While the flu vaccine changes yearly and is therefore the most obvious candidate for evaluating temporal trends in reporting, other vaccines may also change from time to time. For example, the preservative thimerosal was removed from several childhood vaccines in the late 1990s because of theoretical concerns about mercury exposure. Another example was the substitution in 2006 of recombinant human albumin (rHA) for human-derived serum albumin (HSA) in the manufacture of MMR, which eliminated the use of any human-derived substances [17, 26].

Other information external to VAERS will be important to the proper interpretation of findings about heterogeneity of reporting over time. For example, when a highly infectious strain of flu virus is circulating there may be many more cases of flu than in an average year; some cases of flu following vaccination will likely appear in VAERS as individuals may suspect that the vaccine caused them to have the flu. In such a year, there may be increased reports of infections following flu vaccines, which would most likely be due simply to the increased number of flu cases that year.

## Conclusion

In this paper, we propose a rigorous statistical model to detect vaccine safety signals by testing the heterogeneity of reporting rates of given vaccine-event combinations across reporting years using a random effects model with a variance component test. To the best of our knowledge, this is the first method to evaluate *variation of safety signals across years* in a *passive* surveillance database. We proposed a random effects model and formulated the test statistics using composite likelihood functions, which can effectively account for the impact of passive reporting through conditional probability. We found that our proposed likelihood ratio test is powerful in detecting variation of reporting rates over years. Evaluating temporal trends of reporting rates can suggest the potential impact of changes in vaccines on the occurrence of AEs and provide evidence for further investigations.

## Abbreviations

AE: Adverse event
CDC: The centers for disease control and prevention
FDA: The Food and Drug Administration
PT: Preferred term
SOC: System organ class
VAERS: Vaccine adverse event reporting system
